# Effect of γ-tocopherol supplementation on premenstrual symptoms and natriuresis: a randomized, double-blind, placebo-controlled study

**DOI:** 10.1186/s12906-023-03962-5

**Published:** 2023-04-28

**Authors:** Tomoko Higuchi, Tomomi Ueno, Shigeto Uchiyama, Shunji Matsuki, Mariko Ogawa, Kiyoshi Takamatsu

**Affiliations:** 1 Saga Nutraceuticals Research Institute, Otsuka Pharmaceutical Co., Ltd., 5006-5 Yoshinogari, Kanzaki-Gun, Saga, 842-01 Japan; 2 Souseikai Sugioka Memorial Hospital (Currently Souseikai Fukuoka Mirai Hospital) Clinical Research Center, Fukuoka, Japan; 3 Department of Obstetrics and Gynecology, Tokyo Dental College Ichikawa General Hospital, Chiba, Japan

**Keywords:** γ-tocopherol, γ-carboxyethyl hydroxychroman, Premenstrual symptoms, Water retention

## Abstract

**Background:**

This randomized, double-blind, placebo-controlled study aimed to investigate the effects of γ-tocopherol (Toc) supplementation on premenstrual symptoms and natriuresis.

**Methods:**

We enrolled 51 Japanese women with premenstrual symptoms, particularly those who showed increased symptoms induced by water retention during the luteal phase compared with the follicular phase. Premenstrual symptoms were recorded in the first cycle’s postmenstrual follicular phase; physical measurements and urine collection were conducted during the 48-h run-in period. The test supplement, which contained 180 mg of γ-Toc or placebo, was orally administered twice a day for 7 days during the luteal phase of the first and second cycles in a crossover manner. The same evaluation was conducted during the luteal phase, beginning in the morning of the sixth day of supplement administration.

**Results:**

Compared with placebo intake, γ-Toc intake significantly reduced “fatigue” and “irritability/anger” symptoms. Furthermore, compared with placebo intake, γ-Toc intake significantly reduced the thigh circumference. Regarding the “swelling of the legs” and “heavy legs” symptoms and the thigh circumference, the biphasic trend of increasing and decreasing values in the daytime and morning, respectively, during the follicular phase was not observed at the luteal phase with placebo intake. Contrastingly, γ-Toc intake resulted in significantly lower values in the morning than placebo intake. The mean difference in 24-h urinary sodium excretion between γ-Toc and placebo intake was 10.6 mEq (95% confidence interval (CI): -0.1, 21.4, *p* = 0.05, power 55%). Plasma γ-Toc and its metabolite γ-carboxyethyl hydroxychroman (CEHC) levels were significantly higher with γ-Toc intake than with placebo intake. There were no significant between-supplement differences in serum electrolyte levels or cumulative urinary potassium excretion.

**Conclusion:**

γ-Toc intake could effectively alleviate certain premenstrual syndrome symptoms, particularly those related to water retention during the luteal phase. Furthermore, the underlying mechanism may involve the diuretic effect of γ-CEHC, which is a γ-Toc metabolite.

**Trial registration:**

UMIN000047989; registration date: 10/06/2022, retrospectively registered.

**Supplementary Information:**

The online version contains supplementary material available at 10.1186/s12906-023-03962-5.

## Background

In the 1950s, the term premenstrual syndrome (PMS) was introduced for physical and psychological symptoms occurring within 2 weeks before menses, with relief occurring after the onset of the menstrual period [1–3]. According to the American Congress of Obstetricians and Gynecologists (ACOG), the diagnostic criteria for PMS include somatic symptoms, such as abdominal bloating, breast tenderness or swelling, headache, joint or muscle pain, swelling of the extremities, and weight gain, as well as mental symptoms, including angry outbursts, anxiety, confusion, depression, irritability, and social withdrawal [4]. Approximately 95% of Japanese women of reproductive age experience some form of PMS [5].

Although numerous hypotheses regarding the mechanism underlying PMS exist, it remains unclear [1]. In Japan, PMS treatment can be classified as counseling, lifestyle guidance, exercise therapy, and pharmacotherapy. Regarding pharmacotherapy, symptomatic treatment is attempted using diuretics, sedatives, and herbal medicines, as required. Moreover, oral contraceptives containing drospirenone and selective serotonin reuptake inhibitors have been used [6, 7]. Approximately 50% of women present with decreased efficiency and productivity in work and housework [5]; furthermore, periodical PMS symptoms decrease women’s quality of life and result in social losses [8]. However, the concept of PMS is still not widely acknowledged among Japanese women, with Takeda et al. reporting that only 5.3% of women with moderate-to-severe PMS or premenstrual dysphoric disorder receive treatment [5]. Therefore, given the current increase in the entry of women into the workforce, there is a need to increase the treatment alternatives for women whose complaints are not sufficiently severe to require medical treatments or those reluctant to visit a medical institution.

Nutritional supplements, including calcium and vitamin B6, and herbal medicine are effective alternative therapies for mild PMS symptoms [9, 10]. Moreover, a recent study reported a natriuretic effect of γ-carboxyethyl hydroxychroman (CEHC), which is a metabolite of γ-tocopherol (Toc), a natural vitamin E homolog [11]. Kato et al. reported that γ-Toc could effectively treat premenstrual pretibial edema [12]; however, the study did not report increased urinary sodium excretion due to γ-Toc intake and did not examine premenstrual symptoms. Therefore, we aimed to investigate the effects of γ-Toc intake on mitigating premenstrual symptoms and natriuresis.

## Methods

### Study design

This study was a randomized, double-blind, placebo-controlled crossover trial conducted at SOUSEIKAI Sugioka Memorial Hospital in Japan between July 2014 and March 2015. Recruitment was conducted via an online volunteer platform provided by SOUSEIKAI Medical Group. The SOUSEIKAI Hakata Clinic Institutional Review Board approved this study. Written informed consent was obtained from each participant.

### Participants

We enrolled healthy Japanese women who met the following inclusion criteria: age > 20 years, a regular menstrual cycle of 25–38 days with a variation of ≤ 6 days, cyclical onset or worsening of water retention symptoms during the luteal phase, ability to understand the procedures, and provision of informed consent for study participation and intention to comply with the study requirements. The exclusion criteria were as follows: taking any continuous medications or dietary supplements, pregnancy, breastfeeding or intending to become pregnant, current or a history of acute or chronic severe conditions, and clinically relevant findings on physical examination or blood testing.

### Intervention

The intervention was the administration of γ-Toc and a placebo. γ-Toc and placebo were manufactured as capsules and supplied by Eisai Food Chemical (Tokyo, Japan). Each γ-Toc capsule contained 92 mg γ-Toc, 1.7 mg α-Toc, 1.7 mg β-Toc, and 1.0 mg δ-Toc, which was dissolved in vitamin E- stripped corn oil. The placebo capsules only contained vitamin E-stripped corn oil and had the same appearance, size, and color as the γ-Toc capsules. Participants were required to take both test supplements in a crossover fashion, each for 7 days during the luteal phase of one menstrual cycle. Two capsules were taken twice a day, after breakfast and evening meals.

### Study protocol

**Fig. 1:**
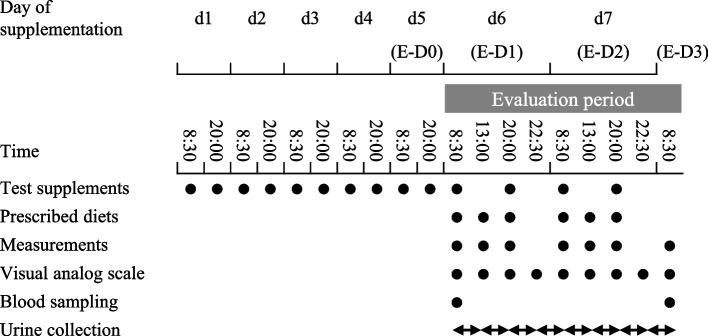
Test schedule

This trial included a screening phase (one menstrual cycle) and two intervention periods (one menstrual cycle each). Participants visited the clinic once during the follicular and luteal phases to screen for eligibility. On the day of the visit, the participants underwent physical measurements and completed questionnaires regarding their physical and menstrual symptoms. Eligible participants were randomly assigned to receive γ-Toc followed by a placebo (group A) or the same supplementation in reverse order (group B) for 7 days in the luteal phase. The washout period was approximately 3 weeks from the completion of the first cycle of luteal phase test supplement intake to the start of the second cycle of luteal phase test supplement intake. Figure 1 shows the test schedule. The evaluation period was a 48-h period from the morning of the sixth day of supplementation (E-D1) to the morning following the seventh day of supplementation (E-D3). Participants remained in the facility from the evening of day 5 (E-D0) to the end of the test. We performed urine collection, blood sampling, and physical measurements during the evaluation period. Furthermore, we administered questionnaires regarding premenstrual symptoms. The participants consumed the same evening meal on E-D0 and received a prescribed diet containing 285.6 mEq sodium/day on E-D1 and E-D2 in each period. In the follicular phase of the first cycle, the same aforementioned procedure was conducted, except for test supplement intake. Participants were prohibited from eating or drinking anything other than the test supplements, prescribed meals, and water. The amount of water consumed was adjusted identically for each individual in each period. On E-D1 and E-D2, the participants were asked to extensively maintain sedentary activity from breakfast to evening meals. We defined the follicular phase as the period from the start of menstruation to 15 days before the next menstruation. Moreover, we defined the luteal phase as the period from 14 days before the next menstruation to the start of the next menstruation. Participants were asked to record and report the dates they recognized menstrual bleeding (menstruation onset) throughout the study period. The test date was set by predicting the follicular and luteal phases based on the previous menstrual cycles; additionally, we confirmed whether the test could be performed at the set date after menstruation onset.

### Assessment of premenstrual symptoms

Regarding the primary outcome measures, the visual analog scale (VAS) scores were used to evaluate the efficacy of γ-Toc against premenstrual symptoms during the evaluation period. The VAS is widely used for questionnaire-based assessment of pain and quantitative analysis of PMS symptoms [13]. At each measurement point, the participants were asked to rate their feeling on a 100-mm scale ranging from “no symptoms” (0 mm) to “severe or extreme symptoms” (100 mm). The VAS was used to evaluate the following 10 items: “swelling of the face,” “swelling of the arms and hands,” “breast tenderness/soreness,” “abdominal bloating,” “swelling of the legs,” “heavy legs,” “fatigue,” “not feeling well/feeling sick,” “irritability/anger,” and “appetite/food craving.” Regarding the secondary outcome measures, the menstrual distress questionnaire (MDQ) was used to evaluate the severity of menstrual symptoms. The MDQ is a 47-item questionnaire developed by Moos [14]. Each item was rated on a six-point (none to severe) scale and categorized into the following eight domains: pain, impaired concentration, behavior change, autonomic reactions, water retention, negative affect, arousal, and control. The VAS was administered in the morning (8:30, before breakfast), before lunch (13:00), in the evening (20:00 before the evening meal), and at bedtime (22:30); moreover, the MDQ was administered in the morning and evening.

### Physical measurement

Regarding the secondary outcome measures, body weight and water content were measured through multi-frequency bioelectrical impedance analysis using InBody 720 (InBody Japan, Tokyo, Japan). Additionally, the waist, thigh, maximum leg, and minimum leg circumferences were measured in the standing position. Regarding the measurement of the thigh and leg circumferences, we determined the height of the measurement site from the floor at the initial measurement, with subsequent measurements taken at the same height. Physical measurements were performed before each meal.

### Measurement of plasma γ-Toc and γ-CEHC levels

Blood samples were collected in the mornings of E-D1 and E-D3. All samples were kept light-protected, refrigerated during the collection period, and frozen at -80 °C until analysis. After removing blinding, plasma γ-Toc levels were measured using high-performance liquid chromatography (HPLC) at an external contract laboratory (SRL, Tokyo, Japan). Plasma γ-CEHC levels were measured by HPLC using a modified version of the method described by Galli et al. [15]. Briefly, plasma unconjugated γ-CEHC was extracted using Oasis HLB μElution 96-well Solid Phase Extraction plates (Waters, Milford, MA, USA) and quantified using a Shimadzu Prominence system equipped with LC-20AD binary gradient pumps (Shimadzu, Kyoto, Japan), a Shimadzu Prominence SIL-20ACHT autosampler, and a coulometric detector (Coularray 5600A detector; ESA, Chelmsford, MA), with the cathodic voltages set at + 200, + 300, + 350, and + 400 mV (Pd as reference). Furthermore, separation was performed on a Capsule Core C18 column (100 mm × 4.6 mm i.d., 2.7 μm; Shiseido, Tokyo, Japan) maintained at 40 °C using an ESA column heater. The solvents for the gradient elution were acetonitrile/H_2_O 2:8 (v/v) containing 50 mM sodium perchlorate, 0.5 mM EDTA-2Na, 0.02% acetic acid (solvent A), and acetonitrile (solvent B). We used the following gradient at a flow rate of 1.0 mL/min: 0–0.1 min, 12.5% B; 0.1–2 min, linear gradient to 18.5% B; 2–4 min, 18.5% B; 4–6 min, linear gradient to 50% B; 6–6.5 min, 50% B and 6.5–10 min, 12.5% B. The detector response showed good linearity within the 0.025–50 μM concentration range. Both the inter- and intra-assay coefficients of variation were < 10%.

### Safety assessment

The medical history of each participant was recorded at the initial screening visit. The safety of the test supplements was assessed by monitoring adverse events, vital signs, clinical laboratory findings, and lengths of menstrual cycles. Additionally, the rate of change in plasma volume from E-D1 morning to E-D3 morning was calculated using hemoglobin and hematocrit values as described by Dill and Costill [16].

### Randomization, allocation, and blinding

Eligible participants were randomly allocated in a 1:1 ratio to receive γ-Toc followed by placebo (group A) or the same supplementation in reverse order (group B), in the order where they were assessed for eligibility by the trial site physician. A computer-generated list of random numbers was used for block randomization (block size = 4). Allocation was performed by the sponsor’s allocator, who was not involved in this study before the enrollment of the first participant. The allocator sealed the allocation list envelope and concealed it in a locked cabinet that only the allocator could open. All participants, personnel, and outcome assessors were blinded until all study data except for plasma γ-Toc and γ-CEHC levels were collected.

### Statistical analysis

Sample size calculations were not performed because of the lack of similar previous studies on the effects of γ-Toc on premenstrual symptoms. Since a double-blind, placebo-controlled report evaluating the efficacy of spironolactone in improving PMS symptoms studied 35 PMS symptomatic patients [17], we decided to conduct this study with a minimum target number of 40 patients, considering dropouts. Statistical analyses were performed using the SAS software version 9.4 (SAS Institute, Cary, NC, USA). The results are presented as the mean and standard error of the mean. Variations in the VAS scores and values of physical measurements were calculated as the difference between the values obtained in the morning of E-D1 and those obtained at each measurement point. Between-supplement differences throughout the evaluation period were analyzed using a general linear mixed model for repeated measures, adjusting carryover effects. Furthermore, between-supplement differences in the values obtained in the mornings of E-D2 or E-D3 were analyzed using a general linear mixed model. Contrastingly, a paired t-test was used to compare values obtained in the morning of E-D2 or E-D3 between the follicular and luteal phases with placebo intake. Statistical significance was set at *P* < 0.05. Efficacy data were analyzed using the per-protocol set (PPS) as the main analysis set. The PPS included all participants who had completed the study as per the protocol. Notably, participants who could not be evaluated within the prescribed menstrual phase were excluded from the PPS.

## Results

### Background characteristics

**Fig. 2:**
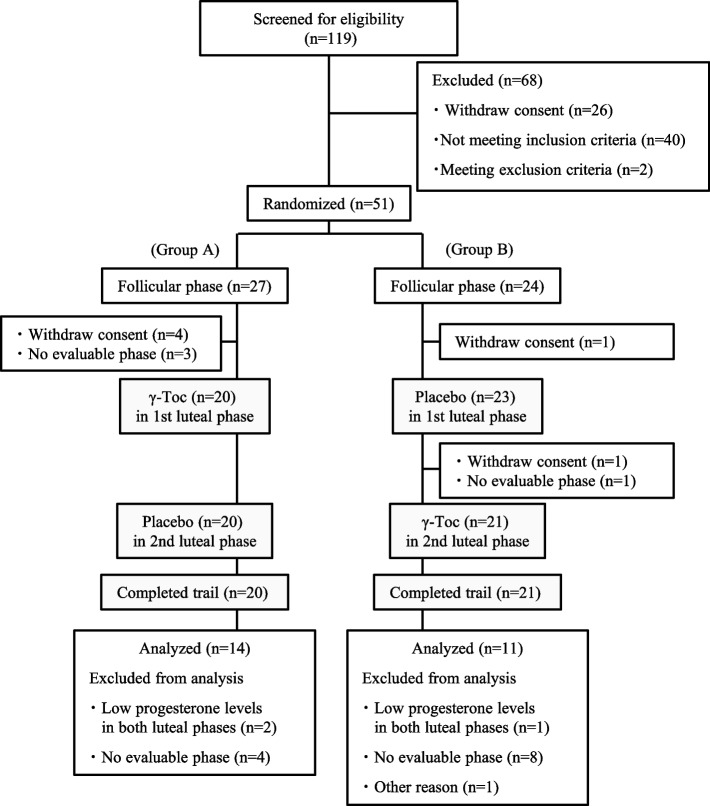
Flow diagram of the study

Figure 2 shows the study flowchart. Recruitment started in June 2014 and ended in August 2014, when the target number of participants was obtained. Potential participants received oral and written information regarding the study from a doctor involved in this study. Among 119 women screened for eligibility, we excluded 68 women (40 did not meet the inclusion criteria, two met the exclusion criteria, and 26 withdrew their consent during the screening period). Subsequently, 51 participants were randomly assigned to groups A and B. As shown in Fig. 2, six participants withdrew consent, and four could not be assessed within the prescribed post-menstruation period; accordingly, they were removed from the study before the start of the second cycle. Of the remaining 25 participants, one took γ-Toc for 9 days for personal reasons; however, the remaining 24 participants consumed the test supplements as prescribed. Finally, 25 participants were included in the efficacy analysis (PPS), including one who used the supplements for 9 days.

Among the 25 participants, the mean age, body mass index, and menstrual cycle length were 27.5 ± 1.2 (range 20–40) years, 21.2 ± 0.5 kg/m^2^, and 29.6 ± 0.4 days, respectively. Table 1 shows the MDQ scores during the follicular and luteal phases within the screening phase. The MDQ scores for all domains apart from arousal were significantly higher in the luteal phase than in the follicular phase. The VAS scores for all 10 items were significantly higher in the luteal phase than in the follicular phase. During γ-Toc and placebo intake, E-D1 in the luteal phase was 6.0 ± 0.5 days and 7.2 ± 0.5 days, respectively, before the onset of the next menstrual period, with no significant between-supplement differences. There were no significant between-supplement differences in the morning serum progesterone and estradiol levels on E-D1 in the luteal phase.

**Table 1:** Baseline characteristics in the follicular and luteal phases

MDQ factors	Follicular phase	Luteal phase	*p*-value
Water retention	2.1 ± 0.4	9.6 ± 0.8	< 0.001
Pain	1.9 ± 0.5	6.9 ± 1.1	< 0.001
Impaired concentration	2.1 ± 0.8	5.6 ± 1.1	0.003
Behavior change	2.0 ± 0.6	5.6 ± 1.0	< 0.001
Autonomic reactions	0.2 ± 0.2	1.1 ± 0.4	0.004
Negative affect	0.8 ± 0.4	5.4 ± 1.0	< 0.001
Arousal	2.1 ± 0.7	1.3 ± 0.5	0.195
Control	0.3 ± 0.2	1.8 ± 0.6	0.005

*MDQ* Menstrual Distress Questionnaire

### Premenstrual symptoms

**Fig. 3:**
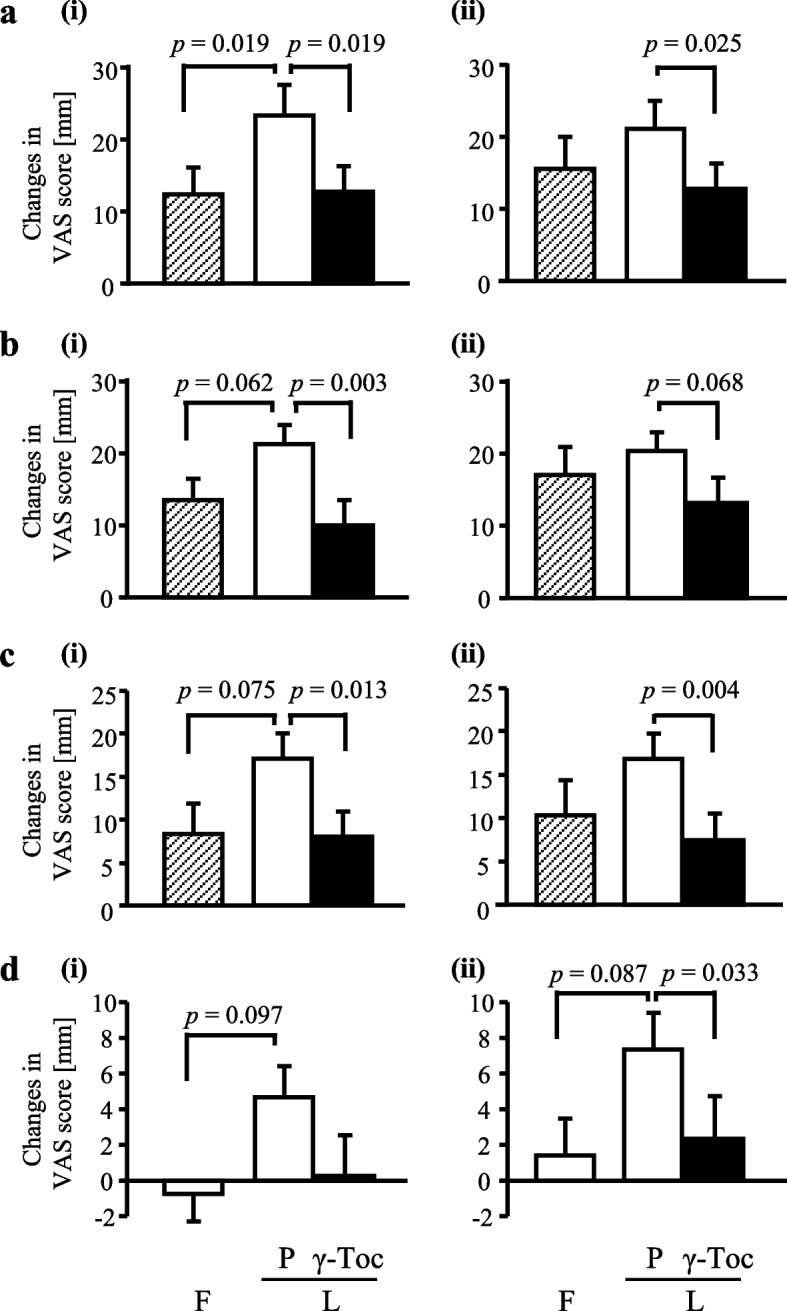
Changes in premenstrual symptom scores: “swelling of the legs” (**a**), “heavy legs” (**b**), “fatigue” (**c**), and “irritability/ anger” (**d**) in the mornings of E-D2 (i) and E-D3 (ii). The data are expressed as the mean ± SEM. The *p*-value is shown in each panel. Shaded columns represent the follicular phase (F), open columns represent placebo (P) intake in the luteal phase (L), and closed columns represent γ-Toc intake in the luteal phase (L)

During placebo intake, the VAS scores for “breast tenderness/soreness” (Supplementary Fig. 1c), “abdominal bloating” (Supplementary Fig. 1d), and “irritability/anger” (Supplementary Fig. 1i) were significantly higher in the luteal phase than in the follicular phase. Moreover, the VAS scores for “fatigue” (Supplementary Fig. 2g) and “irritability/angry” (Supplementary Fig. 2i) were significantly lower with γ-Toc intake than with placebo intake. Furthermore, there were almost significant between-supplement differences in the VAS scores for “heavy legs” (*p* = 0.050, Supplementary Fig. 2f). However, there were no significant between-supplement differences in the variations in the scores for the other items. Contrastingly, regarding the between-phase comparison of variations in symptom severity with placebo intake, “swelling of the legs” (Supplementary Fig. 1e) and “heavy legs” (Supplementary Fig. 1f) showed biphasic variation with peaks in the evenings of E-D1 and E-D2, followed by a decrease in the following morning. However, this biphasic pattern was not as clear in the luteal phase. Accordingly, we performed between-phase and between-supplement comparisons in the luteal phase in the mornings of E-D2 and E-D3 for the following four items: “swelling of the legs,” “heavy legs,” “fatigue,” and “irritability/anger.” During placebo intake, the luteal phase showed significantly higher scores for “swelling of the legs” than follicular phase (Fig. 3). The mean differences in scores for “heavy legs,” “fatigue,” and “irritability/anger” on E-D2 were -7.9 mm (95% confidence interval (CI): -16.2, 0.4, *p* = 0.062, power 46%), -8.7 mm (95% CI: -18.4, 0.9, *p* = 0.075, power 43%), and -5.5 mm (95% CI: -12.0, 1.1, *p* = 0.097, power 38%) (Supplementary Table 1a). Contrastingly, γ-Toc intake resulted in significantly lower scores than the placebo intake for “swelling of the legs,” “heavy legs,” and “fatigue” on E-D2 as well as significantly lower scores for “swelling of the legs,” “fatigue,” and “irritability/anger” on E-D3. The mean difference in scores for “heavy legs” on E-D3 was -7.2 mm (95% CI: -15.0, 0.6, *p* = 0.068, power 46%).

The luteal phase showed significantly higher MDQ scores for “concentration” and “negative affect” than the follicular phase (*p* = 0.006 and *p* = 0.049, respectively. data not shown). During the luteal phase, γ-Toc intake resulted in non-significantly lower MDQ scores for “concentration” than placebo intake (*p* = 0.079).

### Body measurements

**Fig. 4:**
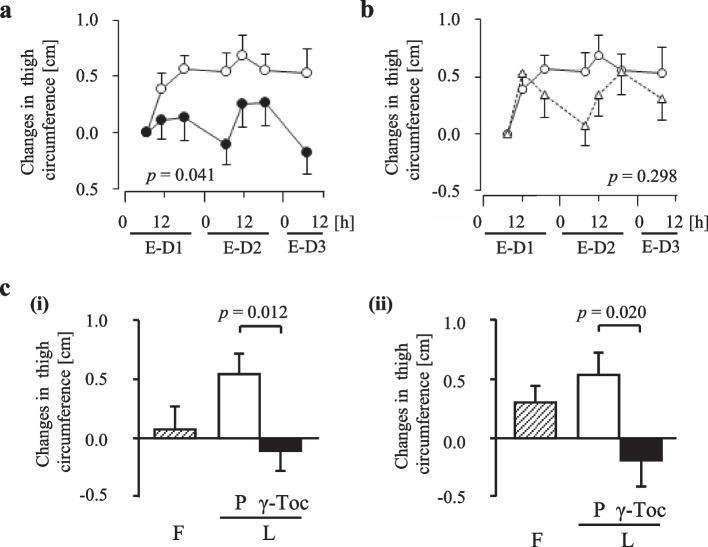
Changes in thigh circumferences: variations during γ-Toc and placebo in the luteal phase during the evaluation period (**a**), variations during the evaluation periods in the follicular and luteal phases (**b**), and values in the morning of E-D2 (**c** [i]) and E-D3 (**c** [ii]). The data are expressed as the mean ± SEM. The *p*-value is shown on each panel. Open triangles (△) represent the follicular phase, open circles (○) represent the luteal phase with placebo intake, and closed circles (●) represent the luteal phase with γ-Toc intake (**a**, **b**). Shaded columns represent the follicular phase (F), open columns represent placebo (P) intake in the luteal phase (L), and closed columns represent γ-Toc intake in the luteal phase (L) (**c**)

Figure 4 shows the changes in thigh circumference. In addition, γ-Toc intake resulted in a significantly lower thigh circumference than the placebo intake (Fig. 4a). During placebo intake, the thigh circumference showed biphasic variation in the follicular rather than luteal phase (Fig. 4b), with a similar pattern being observed with the VAS scores for “swelling of the legs” and “heavy legs”. In the mornings of E-D2 and E-D3, thigh circumference was significantly lower with γ-Toc intake than with placebo intake (Fig. 4c [i] and c [ii]). However, there were no significant between-phase differences in the other parameters.

### Urinary sodium excretion and urine volume

There was no significant between-phase difference in the cumulative urinary sodium excretion and urine volume. In the luteal phase, the mean difference in 24-h urinary sodium excretion between γ-Toc and placebo intake was 10.6 mEq (95% CI: -0.1, 21.4, *p* = 0.052, power 55%). There was no significant between-supplement difference in urine volume (Table 2a). A post hoc analysis was conducted on 17 (68%) participants who showed lower 24-h urinary sodium excretion in the luteal phase than in the follicular phase during placebo intake (Table 2b). There were significant between-phase differences in urinary sodium excretion and urine volume during the evaluation period (data not shown). Furthermore, γ-Toc intake resulted in significantly higher urinary sodium excretion than placebo intake (data not shown); however, there was no significant between-supplement difference in urine volume. Moreover, γ-Toc intake resulted in significantly higher 24-h urinary sodium excretion than the placebo intake (Table 2b). There was no significant between-supplement difference in cumulative urinary potassium excretion (data not shown).

**Table 2:** Changes in cumulative sodium excretion and urine volume during the evaluation period

a) Efficacy analysis (25 participants)			
		24 h	48 h
Urinary sodium excretion (mEq)	Follicular phase	143.9 ± 8.0	359.3 ± 13.6
	* p*-value vs. luteal phase	0.587	0.250
	Luteal phase		
	Placebo intake	139.4 ± 7.1	343.8 ± 13.4
	γ-Toc intake	151.1 ± 7.7	353.1 ± 13.6
	* p*-value vs. placebo intake	0.052	0.464
Urine volume (mL)	Follicular phase	1478 ± 70	3399 ± 112
	* p*-value vs. luteal phase	0.746	0.309
	Luteal phase		
	Placebo intake	1450 ± 67	3283 ± 116
	γ-Toc intake	1480 ± 65	3305 ± 119
	* p*-value vs. placebo intake	0.734	0.981
b) Individuals whose cumulative urinary sodium excretion after 24 h was lower in the luteal phase than in the follicular phase (17 participants)			
		24 h	48 h
Urinary sodium excretion (mEq)	Follicular phase	155.2 ± 9.7	383.2 ± 13.7
	* p*-value vs. luteal phase	0.001	< 0.001
	Luteal phase		
	Placebo intake	129.5 ± 8.6	334.7 ± 17.3
	γ-Toc intake	145.7 ± 9.4	346.0 ± 16.7
	* p*-value vs. placebo intake	0.026	0.630
Urine volume (mL)	Follicular phase	1563 ± 88	3526 ± 136
	* p*-value vs. luteal phase	0.039	0.031
	Luteal phase		
	Placebo intake	1369 ± 87	3235 ± 162
	γ-Toc intake	1433 ± 90	3225 ± 158
	* p*-value vs. placebo intake	0.591	0.436

### Plasma atrial natriuretic peptide (ANP) and aldosterone levels

We performed between-phase and between-supplement comparisons in plasma ANP and aldosterone levels in the efficacy analysis. During placebo intake, there were significantly lower plasma ANP levels in the luteal phase than in the follicular phase in the morning of E-D1 (Table 3). In the luteal phase, γ-Toc intake significantly increased plasma ANP levels in E-D3 mornings, compared with placebo intake. During placebo intake, there were significantly higher plasma aldosterone levels in the luteal phase than in the follicular phase in both E-D1 and E-D3 mornings (Table 3). In the luteal phase, γ-Toc intake significantly decreased plasma aldosterone levels compared with placebo intake in the E-D3 morning.

**Table 3:** Changes in plasma ANP and aldosterone levels during the evaluation period

	Follicular phase (F)	Placebo intake (Luteal phase)	*p*-value vs. F	γ-Toc intake (Luteal phase)	*p*-value vs. Placebo intake
ANP (pg/mL)					
E-D1	27.0 ± 2.8	21.9 ± 1.6	0.041	25.7 ± 2.3	0.059
E-D3	35.5 ± 2.8	32.1 ± 2.5	0.184	42.8 ± 5.4	0.007
Aldosterone (pg/mL)					
E-D1	189.3 ± 16.8	317.6 ± 29.1	< 0.001	278.3 ± 34.9	0.199
E-D3	109.9 ± 8.4	173.0 ± 12.1	< 0.001	138.7 ± 14.2	0.013

*ANP* Atrial natriuretic peptide

### Plasma γ-Toc and γ-CEHC levels

Compared with placebo intake, γ-Toc intake significantly increased plasma γ-Toc levels in the mornings of E-D1 (2.5 ± 0.2 μM vs. 16.3 ± 1.1 μM, *p* < 0.001) and E-D3 (3.4 ± 0.2 μM vs. 17.0 ± 1.0 μM, *p* < 0.001). Similarly, compared with placebo intake, γ-Toc intake significantly increased plasma γ-CEHC levels in the mornings of E-D1 (0.08 ± 0.02 μM vs. 2.87 ± 0.29 μM, *p* < 0.001) and E-D3 (0.11 ± 0.01 μM vs. 3.28 ± 0.26 μM, *p* < 0.001).

### Adverse events and safety

One participant experienced delayed menstruation after γ-Toc consumption; however, no other adverse events were observed. There were no significant between-supplement differences in serum sodium, serum potassium, osmolality, protein concentration, hemoglobin, and hematocrit levels in the mornings of E-D1 and E-D3. Moreover, there was no significant between-supplement difference in the rate of change in plasma volume from E-D1 morning to E-D3 morning.

## Discussion

To the best of our knowledge, this is the first report of γ-Toc intake alleviating physical and psychiatric symptoms in women with water retention symptoms during the luteal phase. Studies have mainly examined the antioxidant and anti-inflammatory effects of γ-Toc [18]; however, its sodium-diuretic effects in humans remain unclear. Additionally, γ-CEHC could induce natriuresis by inhibiting sodium reabsorption via Na^+^/K^+^/2Cl^−^ cotransporters by inhibiting 70 pS potassium channels in the apical membrane of the thick ascending limb of the kidney [11]. Contrastingly, α-CEHC, which is a metabolite of α-Toc, the most widely recognized homolog of vitamin E, lacks a diuretic effect [11]. γ-Toc intake has been reported to exert a diuretic effect on rats fed a high-salt diet [19]. In humans, continuous γ-Toc intake has been reported to increase sodium excretion in healthy adult males [20]. Nevertheless, there has been no study on the efficacy of γ-Toc for premenstrual symptoms.

Regarding PMS symptoms, Takeda et al. reported that > 80% of Japanese women aged 20–49 years present some physical symptoms, including those related to water retention [5]. Symptoms related to water retention during the luteal phase may limit daily activities and affect the motivation to engage in them. Compared with a placebo, spironolactone, which is a diuretic, improves mood and somatic symptoms in women [17]. Moreover, the guidelines of the Royal College of Obstetricians and Gynecologists (Green-top guideline No.48) recommend spironolactone for treating PMS symptoms in women [21]. Overall, reducing symptoms related to water retention may mitigate mental symptoms. Since taking 400 mg γ-Toc for 7 days improved edema [12], we examined whether taking 360 mg γ-Toc for 7 days could improve physical and mental symptoms during the luteal phase.

During the follicular phase, there were biphasic variations in lower extremity symptoms (“swelling of the legs” and “heavy legs”) and thigh circumference, with peaks in the evening and dips in the next morning. This could be attributed to the fact that water stored in the lower limbs due to gravity-induced downward water movement during the day is excreted as urine due to position changes during sleep. Contrastingly, during placebo intake, there was a lesser decrease in lower extremity complaints during the luteal phase than during the follicular phase, even in the morning when the lower extremities were least affected by gravity. Specifically, water retention symptoms in the morning could be a characteristic phenomenon of some women in the luteal phase, which could be treated through γ-Toc intake.

In our study, there were lower and higher plasma ANP and aldosterone levels, respectively, in the luteal phase than in the follicular phase. The observed variations in aldosterone levels are consistent with a previous report by Rosenfeld et al. of increased plasma renin activity and aldosterone levels during the late luteal phase of the menstrual cycle in women with PMS, which contributed to increased fluid retention [22]. Moreover, compared with placebo intake, γ-Toc intake increased and decreased plasma ANP and aldosterone levels, respectively. Therefore, γ-Toc intake may contribute to normalizing fluid distribution disturbances during the luteal phase in women with PMS.

In the post hoc analysis, there was decreased 24-h urinary sodium excretion during placebo intake in the luteal phase than in the follicular phase. Additionally, γ-Toc intake significantly increased sodium excretion compared with placebo intake. These results suggest that γ-Toc moderately mitigates the accumulation of excessive sodium levels in humans, including during the premenstrual period, as reported by Uto et al. in rats [19].

Regarding the safety of γ-Toc ingestion, γ-Toc intake resulted in the menstrual delay in one participant; however, no other adverse events were observed. There were no significant between-supplement differences in blood hemoglobin and hematocrit levels, which suggests that γ-Toc intake does not affect circulating blood volume. Therefore, ingesting 360 mg γ-Toc for 7 days was considered safe.

The strength of this study is that this is the first study to examine the PMS-reducing effects of γ-Toc in a randomized, double-blind, placebo-controlled crossover design. In addition, we prospectively assessed the severity of symptoms during the screening visits and subsequently evaluated the efficacy of the test supplement using the VAS and MDQ scores. Regarding PMS symptoms, there is frequently a mismatch between retrospective complaints and actual symptom patterns; additionally, the ACOG diagnostic criteria require a prospective symptom survey for at least two cycles [4]. The VAS scores, rather than MDQ, revealed a significant reduction in several premenstrual symptoms with γ-Toc intake. Stainer et al. reported that the VAS is a valid, reliable, and sensitive measure for the severity of premenstrual symptoms [13]. Our findings suggest that the VAS is a more sensitive measure for the severity of PMS symptoms than rating scale methods such as the MDQ.

This study had some limitations. First, there was an insufficient sample size. Second, given the relatively complex protocol, we could not confirm the phases using the hormone levels. Third, the number of days until the onset of menstruation following the evaluation period could not be strictly aligned across the individuals or between the groups. Fourth, although we evaluated the efficacy of γ-Toc within a single menstrual cycle, the International Society for Premenstrual Disorders recommends 3 months of interventions for placebo-controlled, parallel-group trials on premenstrual disorders, including PMS [1].

For future recommendations, since there are only two reports regarding the efficacy of γ-Toc intake against water retention during the luteal phase, there is a need for future studies covering multiple cycles with adequate sample size to confirm its efficacy in daily life. Finally, we did not consider the effect of season on the symptoms related to water retention, which should be considered in future studies.

## Conclusions

Supplementation of γ-Toc 360 mg for 7 days during the luteal phase was suggested to alleviate premenstrual somatic and mental complaints, with the underlying mechanism possibly involving the diuretic effect of γ-CEHC. Additionally, intake of γ-Toc 360 mg was well tolerated and safe in premenopausal women. γ-Toc may be useful as a nutritional supplement for mitigating PMS symptoms and improving women’s quality of life. Therefore, additional trials of longer duration and larger sample size are required to confirm these beneficial effects of γ-Toc.

## Supplementary Information

**Additional file 1: Supplemental Fig. 1.** Time-dependent changes in premenstrual symptom scores during the follicular and luteal phases.

**Additional file 2: Supplemental Fig. 2.** Time-dependent changes in premenstrual symptom scores during supplementation.

**Additional file 3: Supplementary Table 1.** 95% confidence interval (CI) of mean differences, *P* values, and power.

## Data Availability

Data used to support the findings of this study are available from the corresponding author upon reasonable request.

## Abbreviations

Toc: Tocopherol
PMS: Premenstrual syndrome
CEHC: Carboxyethyl hydroxychroman
VAS: Visual analog scale
MDQ: Menstrual Distress Questionnaire
ANP: Atrial natriuretic peptide
ACOG: American Congress of Obstetricians and Gynecologists
HPLC: High-performance liquid chromatography
PPS: Per-protocol set
