# Supplementary material for: Effect of γ-tocopherol supplementation on premenstrual symptoms and natriuresis: a randomized, double-blind, placebo-controlled study
Source: BMC Complement Med Ther. 2023 Apr 28;23:136. doi: 10.1186/s12906-023-03962-5 (PMC10148532; doi:10.1186/s12906-023-03962-5)
Supplement: Supplementary file 2 — Additional file 2: Supplemental Fig. 2. Time-dependent changes in premenstrual symptom scores during supplementation. [file 12906_2023_3962_MOESM2_ESM.pptx]

## Slide 1
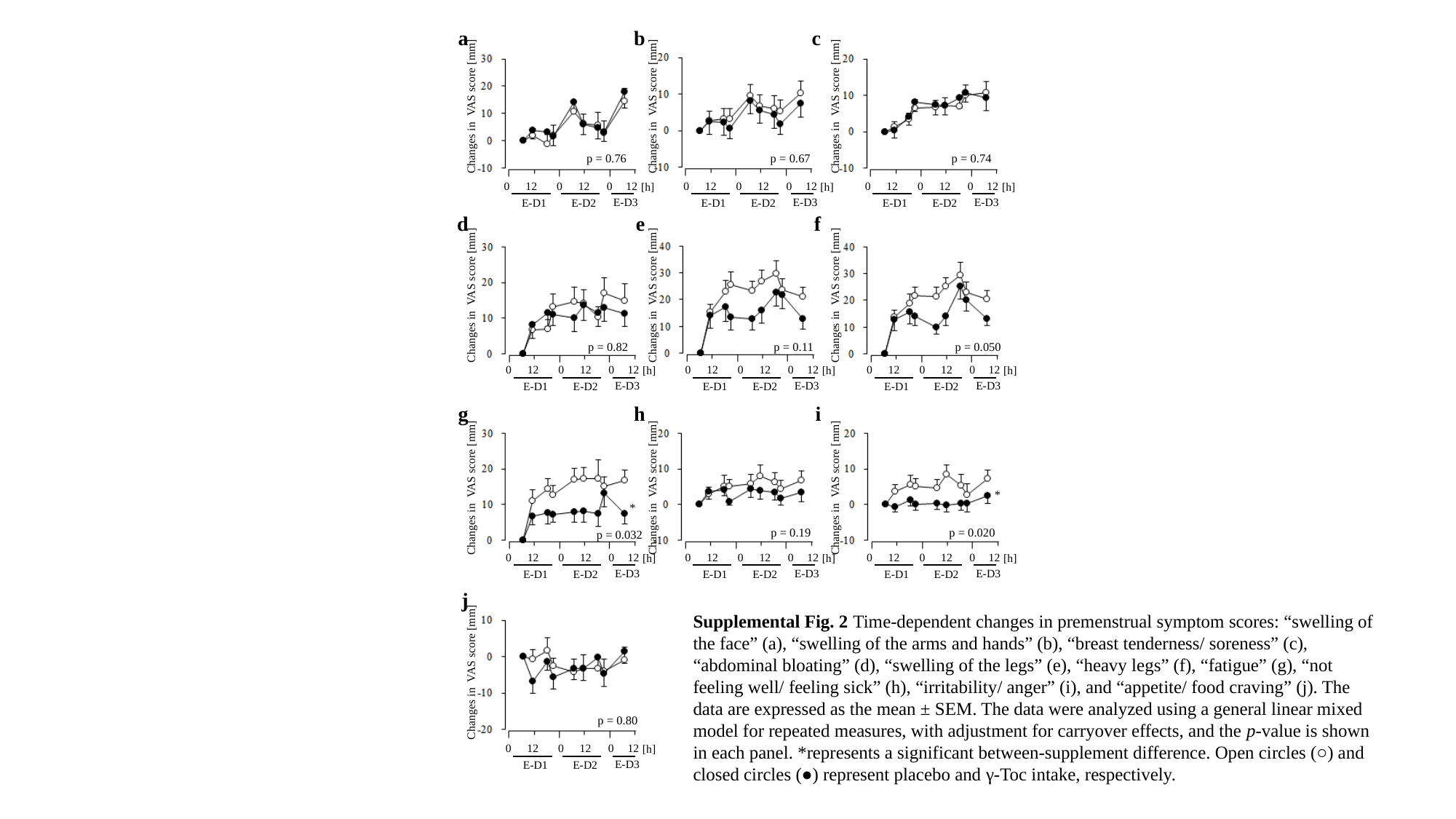

a
b
c
Changes in VAS score [mm]
Changes in VAS score [mm]
Changes in VAS score [mm]
p = 0.76
p = 0.67
p = 0.74
12
12
12
0
0
0
[h]
E-D3
E-D1
E-D2
12
12
12
0
0
0
[h]
E-D3
E-D1
E-D2
12
12
12
0
0
0
[h]
E-D3
E-D1
E-D2
d
e
f
Changes in VAS score [mm]
Changes in VAS score [mm]
Changes in VAS score [mm]
p = 0.82
p = 0.11
p = 0.050
12
12
12
0
0
0
[h]
E-D3
E-D1
E-D2
12
12
12
0
0
0
[h]
E-D3
E-D1
E-D2
12
12
12
0
0
0
[h]
E-D3
E-D1
E-D2
g
h
i
Changes in VAS score [mm]
Changes in VAS score [mm]
Changes in VAS score [mm]
*
*
p = 0.020
p = 0.19
p = 0.032
12
12
12
0
0
0
[h]
E-D3
E-D1
E-D2
12
12
12
0
0
0
[h]
E-D3
E-D1
E-D2
12
12
12
0
0
0
[h]
E-D3
E-D1
E-D2
j
Supplemental Fig. 2 Time-dependent changes in premenstrual symptom scores: “swelling of the face” (a), “swelling of the arms and hands” (b), “breast tenderness/ soreness” (c), “abdominal bloating” (d), “swelling of the legs” (e), “heavy legs” (f), “fatigue” (g), “not feeling well/ feeling sick” (h), “irritability/ anger” (i), and “appetite/ food craving” (j). The data are expressed as the mean ± SEM. The data were analyzed using a general linear mixed model for repeated measures, with adjustment for carryover effects, and the p-value is shown in each panel. *represents a significant between-supplement difference. Open circles (○) and closed circles (●) represent placebo and γ-Toc intake, respectively.
Changes in VAS score [mm]
p = 0.80
12
12
12
0
0
0
[h]
E-D3
E-D1
E-D2
